# A High-Performance Fe@N-S-O-C Nanocomposite-Based Electrochemical Sensor for Dopamine Detection in Pork Samples

**DOI:** 10.3390/foods15162886

**Published:** 2026-08-18

**Authors:** Luyao Wang, Xuelian Wu, Yizi Mahai, Wenjing Ma, Lin Zhou, Jing Zhang, Xinhui Wang, Jing Li

**Affiliations:** 1 College of Food and Biological Engineering, Chengdu University, Chengdu 610106, China; 2 Sichuan Provincial Engineering Research Center of Meat Quality Improvement and Safety Control Technology, Chengdu University, Chengdu 610106, China; 3 Key Laboratory of Fine Chemicals and Surfactants in Sichuan Provincial University, Sichuan University of Science & Engineering, Zigong 643000, China

**Keywords:** dopamine detection, iron-based nanomaterials, electrochemical sensor, food safety, pork samples

## Abstract

Monitoring dopamine (DA) in pork can provide useful information for assessing meat freshness and quality deterioration. In this work, an Fe@N-S-O-C nanocomposite was fabricated as an electrode modifier for DA determination. The Fe@N-S-O-C nanocomposite was synthesized via precipitation followed by calcination using melamine and ferrous sulfate as precursors. The crystal structure, surface chemical composition, and morphology were characterized by XRD, XPS, SEM, and TEM. The Fe@N-S-O-C-modified glassy carbon electrode (Fe@N-S-O-C/GCE) was then evaluated for its electrocatalytic performance toward DA. Under the optimized conditions (pH 6.0), the sensor showed linear responses to DA over 1–65 and 65–220 μM. The sensitivities for these two ranges were 4.357 and 1.685 μA μM^−1^ cm^−2^, respectively, with an LOD of 40 nM. In addition, the Fe@N-S-O-C/GCE showed excellent reproducibility, good repeatability, and strong anti-interference capability against common coexisting substances. After 30 days of storage, 82.74% of the initial current response was retained by the same electrode. Practical applicability of the sensor was verified in pork samples, with recoveries of 95.97–106.73%. These results demonstrate that the Fe@N-S-O-C/GCE sensor offers a reliable and effective platform for DA detection in complex food matrices.

## 1. Introduction

Dopamine (DA), a critical catecholamine neurotransmitter, participates in neural processes underlying memory, cognition, and motor coordination within the central nervous system [1,2]. Disruption of DA homeostasis has been linked to multiple neurological and neuropsychiatric disorders, including depression and other mood disorders, as well as Parkinson’s and Huntington’s diseases [3,4,5,6,7]. Beyond its clinical significance, DA determination has also attracted attention in food analysis. Previous studies have reported the unauthorized administration of DA itself in livestock production to promote lean-meat production and have raised concerns regarding its possible residues in edible tissues [8]. Given that physiological DA concentrations are typically in the sub-micromolar range [9], highly sensitive and accurate analytical methods are required for reliable DA quantification in both biological and food matrices.

To address this need, a variety of analytical techniques are available for DA quantification, such as high-performance liquid chromatography (HPLC), mass spectrometry, and various optical detection methods [10,11,12,13,14,15,16]. While these methods offer high precision, their practical application, especially for on-site food safety screening, is often hindered by high equipment costs, tedious sample pretreatment, and a lack of real-time monitoring capabilities. Electrochemical sensors, in contrast, have attracted considerable interest owing to their intrinsic advantages of fast analytical response, exceptional sensitivity, affordability, and suitability for miniaturized instrumentation [17,18]. However, a major challenge in electrochemical DA sensing remains the interference from co-existing substances, particularly ascorbic acid (AA), which typically exists at much higher concentrations and possesses a similar oxidation potential [19,20]. Consequently, designing electrode materials with exceptional electrocatalytic activity and selectivity is essential.

Iron-based nanomaterials are attractive electrode modifiers because of their natural abundance, low toxicity, and favorable electron transfer properties [21]. In particular, the reversible redox cycling of Fe^2+^/Fe^3+^ pairs on the material surface confers remarkable peroxidase-like and electrocatalytic activities [22,23]. Recent studies suggest that the integration of iron species with heteroatom-doped carbon frameworks (e.g., N or S doping) together with oxygen-containing surface groups may further enhance electrochemical performance by regulating the electronic structure and facilitating target molecule adsorption [24,25,26].

In this study, an Fe@N-S-O-C nanocomposite composed of metallic iron, Fe_4_N, FeS, Fe_3_O_4_, and a carbon matrix was prepared through a simple precipitation method followed by direct calcination. XRD and XPS were used to examine the structure and surface chemistry of the material, while SEM and TEM were used for morphological analysis. The resulting material was subsequently evaluated as an electrode modifier for enzyme-free electrochemical DA detection.

## 2. Materials and Methods

### 2.1. Reagents, Materials and Instruments

Melamine: Adamas Beta (Shanghai) Chemical Reagent Co., Ltd. (Shanghai, China); Ferrous sulfate: Tianjin Damao Chemical Reagent Factory (Tianjin, China); Methanol: Chengdu Nuershi Technology Co., Ltd. (Chengdu, China); Dopamine hydrochloride (DA): Shanghai Macklin Biochemical Technology Co., Ltd. (Shanghai, China); Nafion (5% *w*/*v*): Suzhou Yilong Energy Technology Co., Ltd. (Suzhou, China); Potassium dihydrogen phosphate, potassium hydrogen phosphate, ascorbic acid, phenylalanine (Phe), histidine (His), uric acid (UA), folic acid (FA), tyrosine (Tyr), serine (Ser), and isopropanol: Shanghai Aladdin Biochemical Technology Co., Ltd. (Shanghai, China); Zinc chloride, sodium hydroxide, nicotinic acid (NA), sodium chloride, anhydrous calcium chloride, ferric chloride, magnesium chloride, glucose (Glu), bisphenol A (BPA), and potassium nitrate: Chengdu Kelong Chemical Co., Ltd. (Chengdu, China). Fresh pork samples for the experiment were purchased from Shiling Agricultural and Trade Market in Chengdu, Sichuan, China. All chemicals and reagents of analytical grade used in this study were obtained from commercial suppliers and used without further purification.

TGL-22S Refrigerated Centrifuge: Sichuan Shuke Instrument Co., Ltd. (Chengdu, China); TL1200 Tubular Furnace: Nanjing Boyuntong Instrument Technology Co., Ltd. (Nanjing, China); DX-2700B X-ray Diffractometer (XRD): Dandong Haoyuan Instrument Co., Ltd., (Dandong China); Thermo K-Alpha X-ray Photoelectron Spectrometer (XPS): Thermo Fisher Scientific K-Alpha, Waltham, MA, USA; Zeiss Gemini 300 Scanning Electron Microscope (SEM): Carl Zeiss Microscopy GmbH, Jena, Germany; JEOL-2100F Transmission Electron Microscope (TEM): JEOL, Ltd., Tokyo, Japan; and a CHI660e Electrochemical Workstation: Shanghai Chenhua Co., Ltd., Shanghai China.

### 2.2. Preparation of Fe@N-S-O-C Nanocomposites

To prepare the precursor, a suspension containing 3.0 g of melamine in 120 mL of methanol/water (1:2, *v*/*v*) was stirred at 80 °C for 30 min. Separately, a homogeneous ferrous sulfate solution was prepared by dissolving 4.5 g of ferrous sulfate in 40 mL of deionized water with stirring at 45 °C for 5 min. The resulting iron solution was then rapidly introduced into the melamine suspension. The combined mixture was maintained under stirring at 80 °C for a further 2 h, during which a black colloidal suspension formed. Upon completion of the reaction, the black suspension was subjected to centrifugation at 7000 rpm for 5 min to isolate the solid product. The recovered precipitate was then thoroughly washed with ultrapure water four times to remove residual soluble species. The washed solid was subsequently dried under vacuum at 70 °C overnight, yielding the precursor product for the subsequent thermal treatment. Ultimately, the obtained precursor underwent thermal calcination at 900 °C for 2 h under a continuous argon atmosphere. After calcination, the resulting material was collected and designated as the Fe@N-S-O-C nanohybrid.

### 2.3. Characterization of Fe@N-S-O-C Nanocomposites

The crystallographic phase of the target materials was determined using XRD with a copper target (*λ* = 0.15418 nm), operating at 40 kV and 30 mA. The surface element composition was analyzed via XPS. Furthermore, the microscopic morphological features of the prepared composites were investigated in detail via SEM and TEM.

### 2.4. Preparation of Fe@N-S-O-C/GCE and Electrochemical Testing

**Scheme 1:**
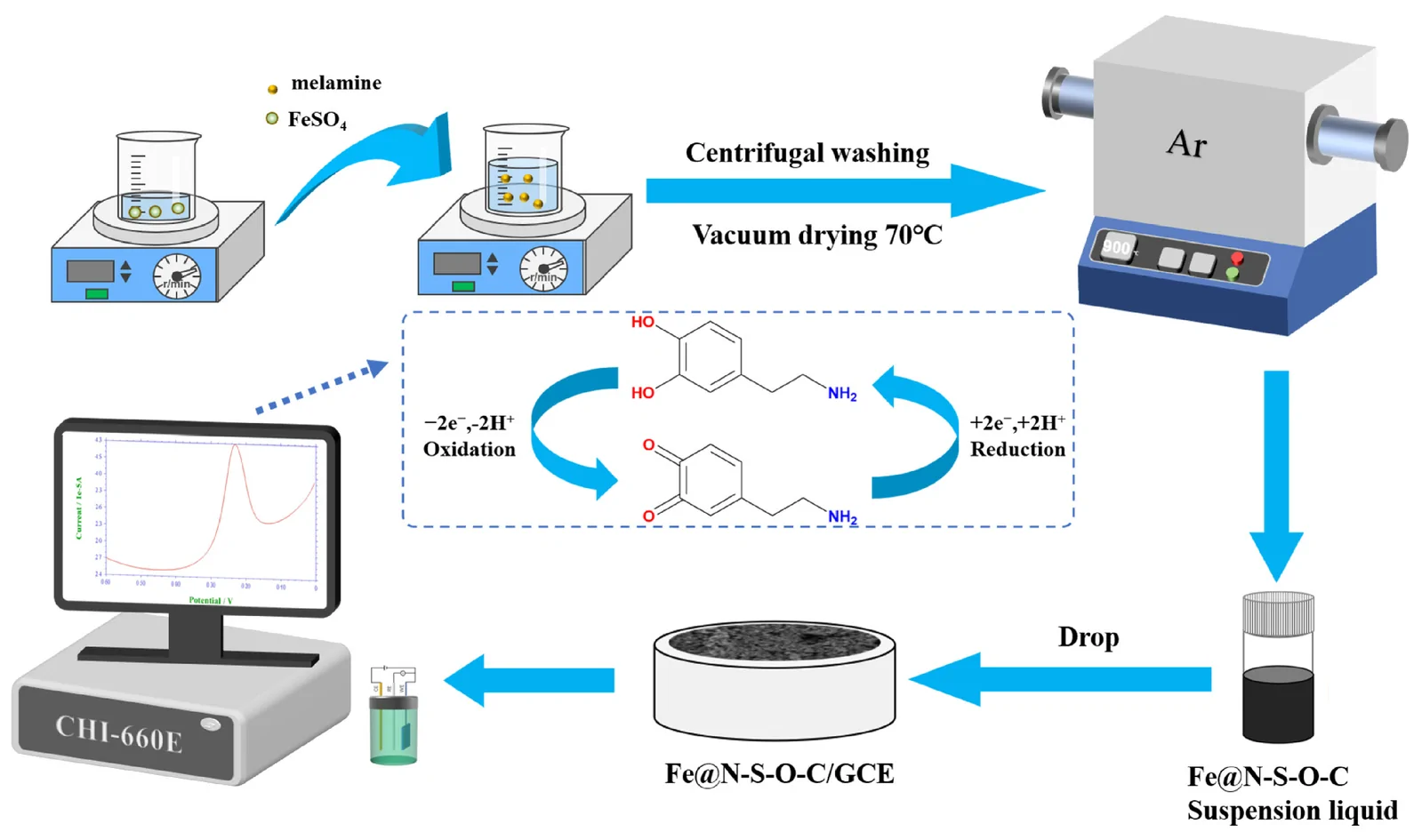
Schematic illustration of the synthetic route for the Fe@N-S-O-C nanocomposite and the fabrication of the Fe@N-S-O-C/GCE electrode for DA sensing.

Prior to the electrode preparation procedure, a bare glassy carbon electrode (GCE) with a diameter of 3 mm was subjected to sequential polishing on a polishing cloth using 0.3 μm and 0.05 μm alumina powder pastes. Following the polishing step, the electrode was ultrasonically rinsed successively with ultrapure water, anhydrous ethanol, and ultrapure water again to remove any residual contaminants. A specific amount of Fe@N-S-O-C nanocomposite material was mixed with 25 μL of Nafion in a 500 μL isopropanol–water mixture (Visopropanol: Vwater = 1:4) and ultrasonicated to form a homogeneous suspension. Subsequently, 3.5 μL of the freshly prepared dispersion was carefully withdrawn with a micropipette and deposited onto the central region of the pre-cleaned GCE surface. The coated electrode was then allowed to dry naturally overnight under ambient conditions. The resulting modified electrode was designated as Fe@N-S-O-C/GCE (Scheme 1). All electrochemical measurements were conducted at ambient temperature using a CHI660E workstation equipped with a conventional three-electrode system. The Fe@N-S-O-C/GCE (or bare GCE) was employed as the working electrode (WE), while a platinum wire (*d* = 3 mm) and an Ag/AgCl electrode were used as the counter electrode (CE) and reference electrode (RE), respectively. The electrochemical analytical methods applied in this work included cyclic voltammetry (CV), differential pulse voltammetry (DPV), and chronocoulometry (CC).

### 2.5. Determination of DA Content in Actual Samples

Fresh pork samples were obtained from a local market and pretreated using the zinc chloride protein precipitation procedure [27]. Briefly, 4.0 g of pork tissue was thoroughly homogenized with 10 mL of 0.2 M phosphate-buffered saline (PBS) to form a uniform slurry. Subsequently, 10 mL of 1 M ZnCl_2_ solution was added, and the mixture was stirred continuously for 10 min to facilitate protein precipitation. The resulting suspension was centrifuged at 5000 rpm for 15 min at 4 °C. The collected supernatant was subsequently clarified using a 0.22 μm membrane filter. The combined precipitation, centrifugation, and filtration procedures were used to reduce proteins, including myoglobin and associated heme species, as well as particulate matter, thereby minimizing potential heme-related interference and electrode fouling during electrochemical analysis. The resulting filtrate was used directly for subsequent DA measurements.

## 3. Results and Analysis

### 3.1. Microstructure and Chemical Composition of Fe@N-S-O-C Nanocomposites

**Figure 1:**
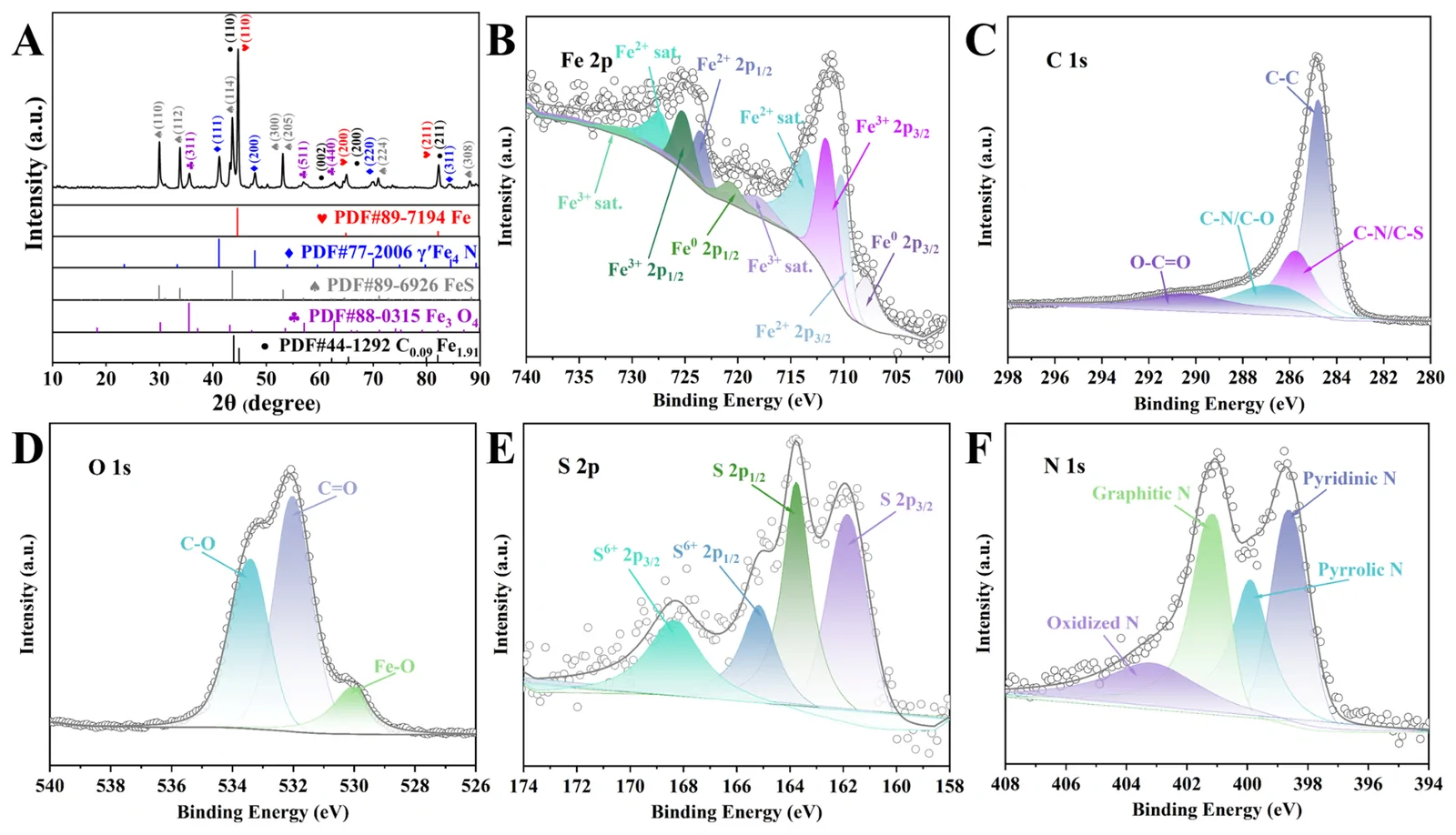
(**A**) XRD pattern of the Fe@N-S-O-C nanohybrid. (**B**–**F**) The corresponding high-resolution XPS spectra showing the characteristic peaks of (**B**) Fe 2p, (**C**) C 1s, (**D**) O 1s, (**E**) S 2p, and (**F**) N 1s.

The crystal structure and phase composition of the prepared nanocomposite were examined by XRD, as shown in Figure 1A. Prominent diffraction peaks appearing at 44.7°, 65.0°, and 82.3° correspond closely to the (110), (200), and (211) planes of metallic Fe (JCPDS: 89-7194), respectively [28]. Distinctive reflections located at 41.2°, 47.9°, 70.1°, and 84.8° are indexed to the (111), (200), (220), and (311) crystallographic planes of the standard γ′-Fe_4_N phase (PDF No. 77-2006) [29]. And the diffraction peaks detected at 29.9°, 33.8°, 43.7°, 53.2°, 71.2°, and 88.5° are indexed to the (110), (112), (114), (300), (205), (224), and (308) planes of hexagonal FeS (JCPDS: 89-6926) [30]. There are some diffraction peaks that match Fe_3_O_4_ (JCPDS: 88-0315) [31], with prominent peaks at 35.5°, 57.1°, and 62.7° corresponding to the (311), (511), and (440) planes. Moreover, several low-intensity peaks at 43.9°, 44.9°, 65.3°, and 82.1° are attributable to the (101), (110), (200), and (211) planes of the iron carbide C_0.09_Fe_1.91_ (JCPDS: 44-1292) [32]. The XRD profile of the Fe@N-S-O-C nanocomposites confirms the presence of characteristic peaks corresponding to Fe, Fe_4_N, FeS, Fe_3_O_4_, and C_0.09_Fe_1.91_, validating the successful synthesis of the target material.

XPS characterization was subsequently performed to gain further insight into the chemical composition and valence states of the nanocomposite. According to the survey spectrum shown in Figure S1, the fabricated composite was confirmed to consist of five elemental species, namely C, Fe, O, N, and S, which is consistent with the crystallographic information obtained from XRD analysis. The high-resolution Fe 2p spectrum (Figure 1B) shows Fe 2p_3/2_ components at 707.8 eV, 710.09 eV, and 711.52 eV, assigned to Fe^0^, Fe^2+^, and Fe^3+^, respectively. The corresponding Fe 2p_1/2_ peaks are observed at 720.6 eV, 723.41 eV, and 725.12 eV. Furthermore, the peaks at 713.5 eV and 727.3 eV are identified as the satellite peaks of Fe^2+^, while those at 718.08 eV and 730.88 eV are assigned to the satellite peaks of Fe^3+^ [28].

The C 1s spectrum (Figure 1C) displays four distinct peaks located at 284.8 eV (C-C), 285.7 eV (C-N/C-S), 286.7 eV (C-N/C-O), and 290.5 eV (O-C=O) [33,34]. The O 1s spectrum (Figure 1D) reveals three oxygen species at binding energies of 530.2 eV [35], 532.0 eV, and 533.4 eV [36]. In the high-resolution S 2p spectrum (Figure 1E), the broad envelope can be deconvoluted into S 2p_3/2_ and S 2p_1/2_ doublets. The peak positioned at 161.9 eV is attributed to the S 2p_3/2_ component of the Fe-S bond, accompanied by a satellite peak at 163.7 eV. The associated S 2p_1/2_ peak is observed at 165.2 eV. Additionally, the high binding energy peak at 168.3 eV reveals the presence of highly oxidized sulfur, which is assigned to the -C-O-S- bond [28]. The high-resolution N 1s spectrum (Figure 1F) can be deconvoluted into four characteristic peaks at binding energies of 398.6, 399.9, 401.1, and 403.2 eV, which are assigned to pyridinic N, pyrrolic N, graphitic N, and oxidized N species, respectively [37]. Pyridinic N, which is mainly distributed at the edge or defect sites of the graphitic framework, contributes to the aromatic π-conjugated system while preserving an accessible lone pair of electrons, thus providing favorable coordination sites for metal species. Meanwhile, pyrrolic N and graphitic N act as π-electron donors, promoting band-gap narrowing of the carbon framework and thereby improving the electrochemical performance of the material [38]. The XPS results confirm that the surface of the as-synthesized Fe@N-S-O-C nanocomposite contains abundant Fe^3+^ and Fe^2+^ species, along with diverse carbon, nitrogen, sulfur, and oxygen bonds. These multivalent iron species contribute favorably to the electrical conductivity and electrocatalytic activity of the nanocomposite.

**Figure 2:**
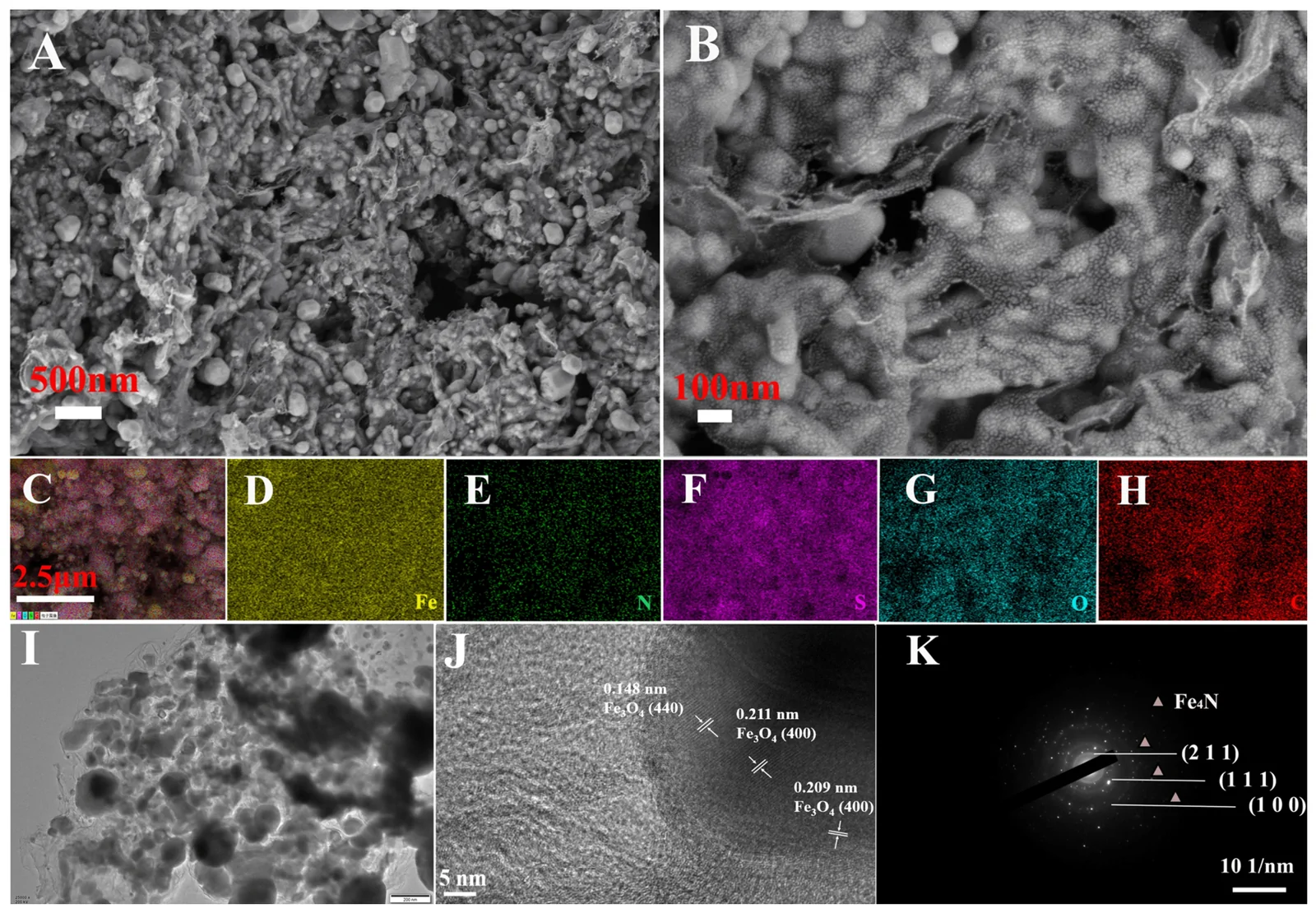
(**A**,**B**) Representative SEM images of the Fe@N-S-O-C nanohybrid at different magnifications. (**C**) Elemental mapping image showing the uniform distribution of (**D**) Fe, (**E**) N, (**F**) S, (**G**) O, and (**H**) C throughout the composite. (**I**) TEM image, (**J**) HRTEM image showing the lattice fringes, and (**K**) the corresponding SAED pattern of the as-prepared nanohybrid.

The microstructure and morphological characteristics of the Fe@N-S-O-C nanocomposites were analyzed by using SEM and TEM. The SEM images (Figure 2A,B) indicate that the Fe@N-S-O-C composite possesses a rough and wrinkled morphology with irregularly stacked sheet-like structures. Numerous nanoparticles or nanoscale protrusions are distributed over the surface, forming a relatively loose and porous architecture. Figure 2C further reveals a tight interconnection between the spherical particles and the flake-like matrix, indicating its stable structure. Such morphological features may contribute to an enlarged electrochemically accessible interface and may therefore be beneficial for electrochemical sensing applications [39]. As illustrated in Figure 2 D–H, the elements Fe, N, S, O, and C are uniformly distributed throughout the material. This homogeneity enhances the electrochemical activity and energy storage performance of the material, while also reducing the internal resistance of the electrode and mitigating inhomogeneities in electrochemical reactions [40]. Visual inspection of Figure 2I–K further confirms that the sheet-like constituent of the Fe@N-S-O-C nanocomposite accommodates spherical nanoparticles characterized by a broad size distribution, whereby the overall morphological landscape is primarily defined by these spherical units. Regarding the crystallographic details, the measured lattice spacing of approximately 0.148 nm is consistent with the (440) plane of Fe_3_O_4_, whereas the two measured values of 0.209 and 0.211 nm are consistent with the (400) plane, according to PDF No. 88-0315. These assignments agree with the XRD results.

### 3.2. Electrochemical Behavior

**Figure 3:**
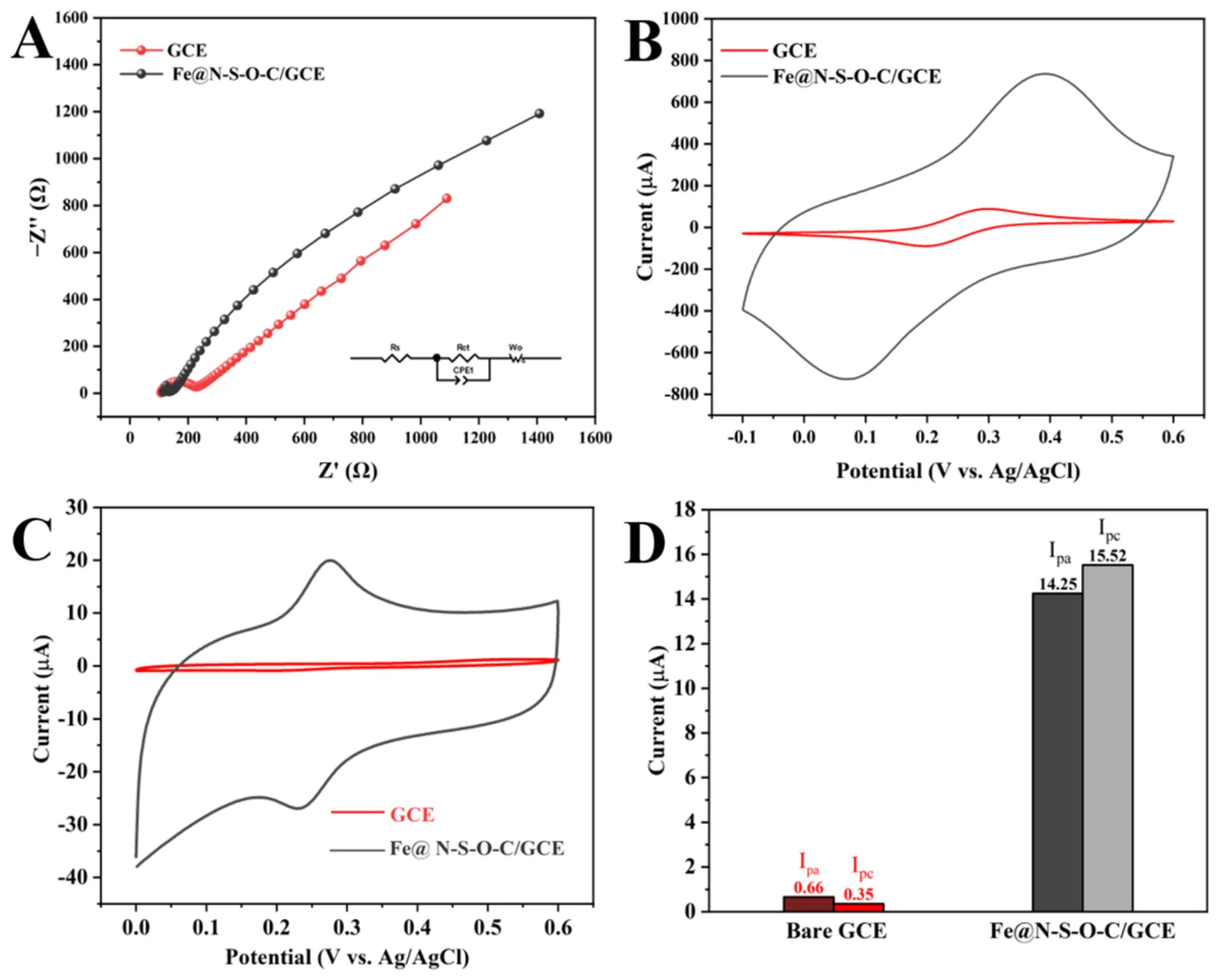
(**A**) Nyquist impedance spectra of the bare GCE and Fe@N-S-O-C/GCE, with the analogous equivalent circuit presented in the inset. (**B**) CV recorded for the bare GCE and Fe@N-S-O-C/GCE at scan rate of 50 mV s^−1^. (**C**) CV profiles acquired in PBS (pH 6.0) containing 20 μM DA for both electrodes, and (**D**) comparison of the redox peak currents of the bare GCE and Fe@N-S-O-C/GCE.

The interfacial electrochemical characteristics of the bare GCE and the Fe@N-S-O-C-modified electrode were investigated by EIS and CV using 5 mM [Fe(CN)_6_]^3−^/^4−^ in 0.1 M KCl as the supporting electrolyte. As shown in the Nyquist plots (Figure 3A), the fitted charge transfer resistance (*R_ct_*) decreased markedly from 128.65 Ω for the bare GCE to 23.31 Ω for the Fe@N-S-O-C/GCE, indicating that the nanocomposite effectively lowers the electron-transfer barrier and accelerates interfacial kinetics. CV measurements (Figure 3B) further confirmed this result. The modified electrode exhibited significantly enhanced background currents and well-defined reversible redox peaks. Consistent with the EIS results, this improved performance is attributed to the low charge-transfer resistance and the possible contribution of Fe-containing sites in the Fe@N-S-O-C material, which facilitate interfacial electron transfer and DA oxidation, respectively [41].

To investigate the electrocatalytic activity of the prepared Fe@N-S-O-C nanohybrid toward DA, the bare GCE and Fe@N-S-O-C/GCE were comparatively examined. As shown in Figure 3C, the bare GCE exhibited relatively weak redox activity toward DA. In contrast, the Fe@N-S-O-C/GCE displayed significantly enhanced redox peak currents, indicating its superior electrocatalytic performance toward DA oxidation and reduction. Upon the addition of DA, the bare GCE produced an oxidation peak at 0.55 V with a peak current of only 0.66 μA. In contrast, a well-defined oxidation peak was observed at 0.28 V for the Fe@N-S-O-C/GCE, with a peak current of 14.25 μA, approximately 21 times that of the bare GCE. Similarly, a reduction peak appeared at 0.19 V for the bare GCE with a current of merely 0.35 μA, whereas the Fe@N-S-O-C/GCE exhibited a distinct reduction peak at 0.23 V with a peak current of 15.52 μA, about 44 times higher than that of the bare GCE (Figure 3D). These results indicate that the Fe@N-S-O-C nanocomposite significantly enhances the electrocatalytic performance of the sensor toward DA. The enhanced electrocatalytic performance of the sensor may be attributed to the enlarged electrochemically active surface area and improved interfacial electron-transfer behavior of the Fe@N-S-O-C/GCE, which facilitate redox kinetics and lead to increased peak currents [42].

**Figure 4:**
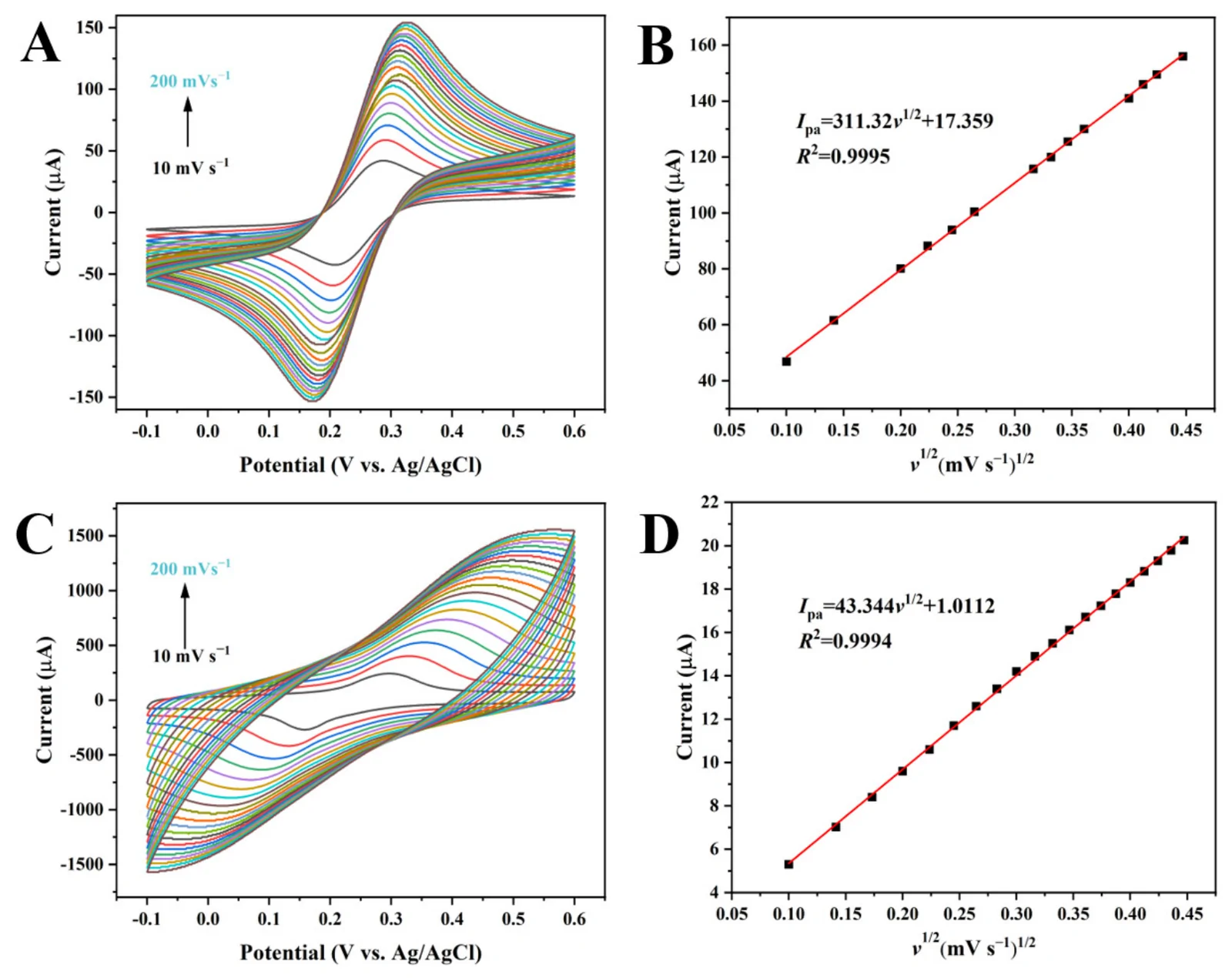
(**A**) and (**C**) display the CV profiles of the bare GCE and Fe@N-S-O-C/GCE, respectively, recorded within a scan rate range of 10–200 mV s^−1^. (**B**) and (**D**) show the corresponding linear regression graphs plotting the anodic peak current (*I*_pa_) against the square root of the scan rate (*v*^1/2^).

The electrochemical active surface areas (ECSAs) of the bare GCE and Fe@N-S-O-C/GCE were evaluated using cyclic voltammetry in 5 mM [Fe(CN)_6_]^3−^/^4−^ containing 0.1 M KCl. As shown in Figure 4A,C, CV curves were recorded at scan rates ranging from 10 to 200 mV s^−1^. Both electrodes exhibited well-defined redox responses, and the peak currents increased linearly with the scan rate, indicating the good electrochemical reversibility of the [Fe(CN)_6_]^3−^/^4−^ redox couple. Moreover, the anodic peak current (*I*_pa_) showed an excellent linear relationship with the square root of the scan rate (*v*^1/2^) (*R*^2^ > 0.9990), suggesting that the electrochemical process was diffusion-controlled. Utilizing the Randles–Sevcik equation (1) [43], the ECSA values were derived to be 0.089 cm^2^ for the bare GCE and 1.11 cm^2^ for the Fe@N-S-O-C/GCE. Particularly, the Fe@N-S-O-C/GCE featured an ECSA that was approximately 12.3-fold larger than that of the bare GCE. The observed prominent enhancement in electrochemical accessibility arises primarily from the hierarchically porous architecture and the inherently high surface area of the Fe@N-S-O-C nanocomposite. These morphological merits not only create a multitude of accessible catalytic sites, but also accelerate interfacial charge transport, which together lead to the superior sensing performance.

(1) $$I_{pa}=2.69\times 10^{5}AD^{1/2}n^{3/2}v^{1/2}C_{0}$$

where *I*_pa_ is the anodic peak current (A); *A* is the electrochemically active surface area (cm^2^) of the electrode; *D* is the diffusion coefficient of 5 mM [Fe(CN)_6_]^3−^/^4−^ in 0.1 M KCl; n is the number of electrons transferred in the redox reaction; *v* is the scan rate (mV s^−1^); and *C*_0_ is the concentration of the redox probe.

**Figure 5:**
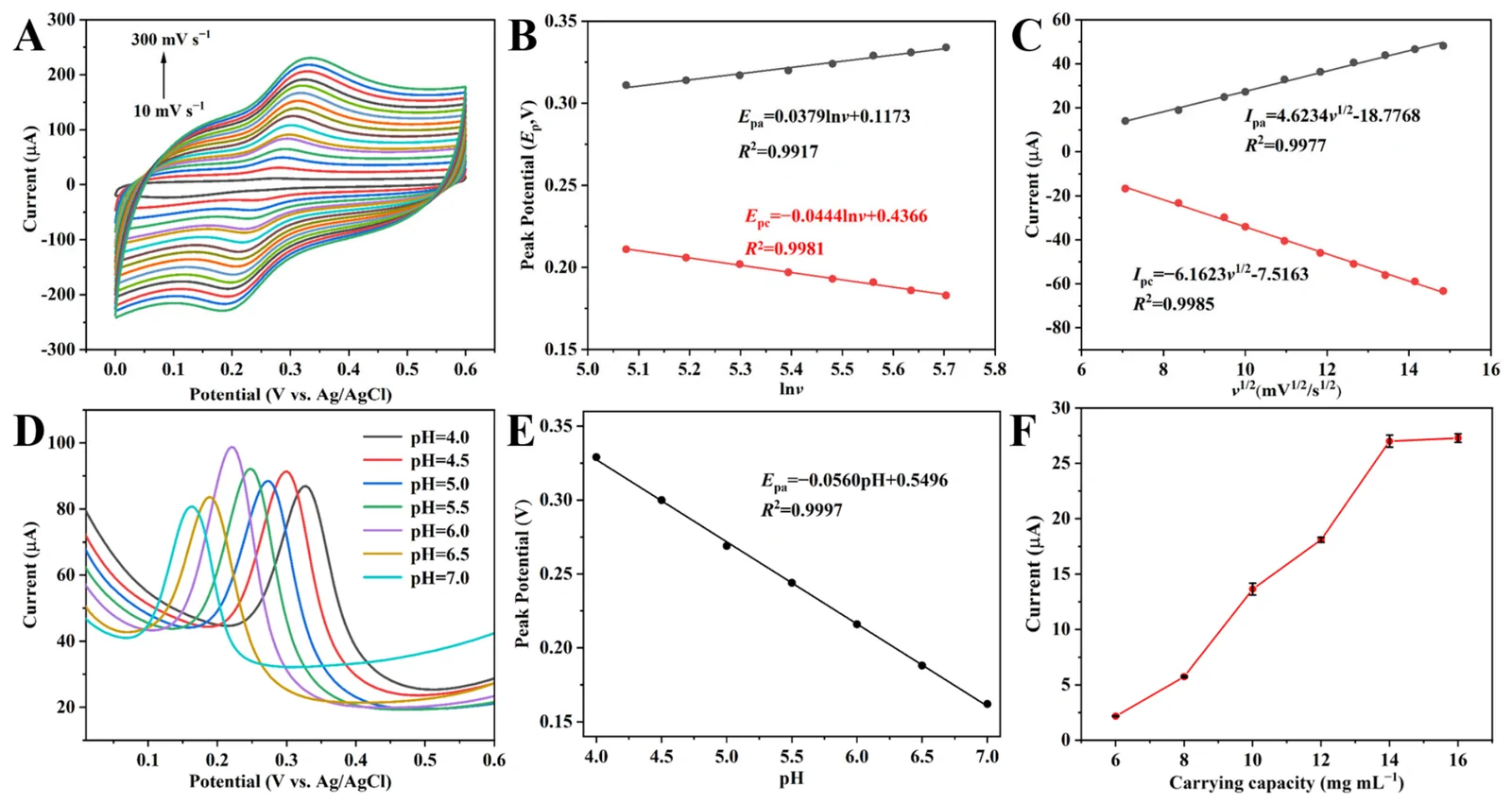
(**A**) CV of 20 μM DA at Fe@N-S-O-C/GCE in 0.1 M PBS (pH = 6.0) at varying scan rates (10–300 mV/s). (**B**) Linear fitting of peak potential (*E*_pa_) versus the natural logarithm of scan rate. (**C**) Linear fitting of peak current (*I*_pa_) versus the square root of scan rate (*v*^1/2^). (**D**) DPV curves of DA at the Fe@N-S-O-C/GCE in PBS with varying pH values (4.0–7.0). (**E**) The corresponding linear plot of the anodic peak potential (*E*_pa_) versus pH. (**F**) Effect of the Fe@N-S-O-C loading concentration on the anodic peak current of DA.

To investigate the effect of the scan rate, CV measurements were performed at scan rates ranging from 10 to 300 mV s^−1^, as shown in Figure 5A. As the scan rate increased, both anodic and cathodic peak currents gradually increased, accompanied by systematic shifts in the corresponding peak potentials. The anodic peak shifted toward more positive potentials, whereas the cathodic peak shifted toward more negative potentials. Further analysis (Figure 5B) revealed that the anodic (*E*_pa_) and cathodic (*E*_pc_) peak potentials exhibited a strong linear correlation with the natural logarithm of the scan rate (ln*v*). The corresponding linear regression equations are expressed as follows: *E*_pa_ = 0.0379ln*v* + 0.1173 (*R*^2^ = 0.9917), *E*_pc_ = −0.0444ln*v* + 0.4366 (*R*^2^ = 0.9981). According to the Laviron equations for a surface-controlled electrode process [44]:

(2) $$E_{pa}=E^{\circ }+\frac{RΤ\ln\upsilon }{(1-\alpha )nF}$$

(3) $$E_{PC}=E^{\circ }-\frac{RΤ\ln\nu }{\alpha nF}$$

where *F* is the Faraday constant, *R* is the universal gas constant, and *T* is the absolute temperature (in K). Based on the slopes of the *E*_p_ versus ln*v* plots, the electron transfer coefficient (*α*) and the number of transferred electrons (*n*) were calculated to be approximately 0.46 and 2, respectively. Additionally, by plotting the peak currents (*I*_p_) against *v*^1/2^ (Figure 5C), strong linearity was observed for both anodic and cathodic responses. The corresponding linear regression equations were respectively fitted as: *I*_pa_ = 4.6234*v*^1/2^ − 18.7768 (*R*^2^ = 0.9977) and *I*_pc_ = −6.1623*v*^1/2^ + 7.5163 (*R*^2^ = 0.9985). These findings strongly indicate that the electrochemical redox process of DA at the modified electrode is predominantly a diffusion-controlled mechanism [45].

### 3.3. Optimization of Sensing Conditions

**Scheme 2:**
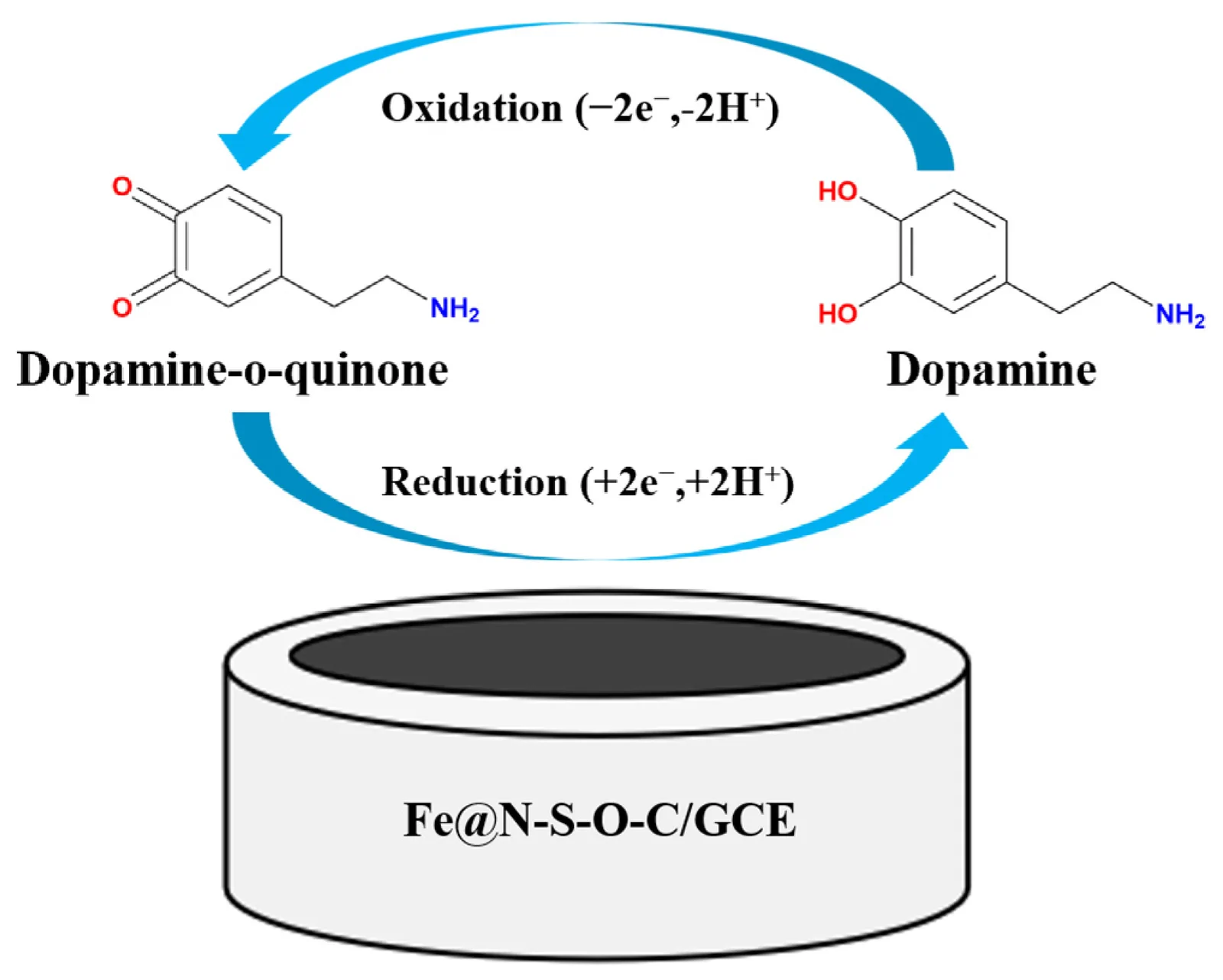
Schematic illustration of the proposed two-electron/two-proton electrochemical redox mechanism of DA at the Fe@N-S-O-C/GCE.

The influence of pH on the electrochemical response of DA at the Fe@N-S-O-C/GCE was investigated using differential pulse voltammetry (DPV) in 0.1 M PBS with pH values ranging from 4.0 to 7.0. As depicted in Figure 5D, both the peak current magnitude and the corresponding potential exhibited a considerable dependence on the electrolyte pH. Upon increasing the pH, the anodic peak potential (*E*_pa_) underwent a progressive shift toward more negative potentials, indicating that protons are directly involved in the oxidation reaction of DA. Furthermore, an excellent linear correlation was observed between *E*_pa_ and pH (Figure 5E), with the linear regression equation expressed as *E*_pa_ = −0.0556pH + 0.5496 (*R*^2^ = 0.9997). In accordance with the Nernst equation [46]:

(4) $$E_{\mathrm{pa}}\;=\;E^{0}\;-\;(\frac{2.303mRT}{nF})\mathrm{pH}$$

in which *E*^0^ signifies the standard electrode potential, while m and *n* correspond to the number of protons and electrons participating in the reaction, respectively, and *R*, *T*, and *F* retain their usual physical constants. Based on the experimental slope of −55.6 mV/pH, the proton-to-electron ratio (*m*/*n*) was estimated to be approximately 0.94, suggesting that approximately equal numbers of protons and electrons are involved in the electrochemical reaction [47]. Combined with the Laviron analysis, which yielded *n* ≈ 2, these results suggest that DA undergoes a two-electron/two-proton oxidation at the Fe@N-S-O-C/GCE to form dopamine-o-quinone, while the reverse reduction converts dopamine-o-quinone back to DA, as illustrated in Scheme 2. Regarding analytical sensitivity, the electrode exhibited the maximum anodic peak current at pH 6.0, whereas the current decreased significantly at higher pH values. Therefore, pH 6.0 was selected as the optimal condition for subsequent electrochemical detection.

The loading amount of the modifier is a critical factor influencing the electrochemical detection performance. To optimize this parameter, DPV was employed to record the anodic peak currents of DA using various Fe@N-S-O-C suspension concentrations (ranging from 6.0 to 16.0 mg mL^−1^). As demonstrated in Figure 5F, the current response revealed a continuous ascent with the loading concentration, culminating in an optimal response at 14.0 mg mL^−1^. However, an excessive loading surpassing this critical value triggered an apparent decline in the detectable signal. Such an observation can be plausibly interpreted by the formation of an excessively dense film at higher loadings, which may cause the structural collapse of the material’s three-dimensional architecture or severe aggregation [48]. Such structural degradation hinders mass and electron transfer, thereby reducing the effective electroactive surface area. Consequently, an optimal suspension concentration of 14.0 mg mL^−1^ was selected for the preparation of the modified electrode in all subsequent experiments.

### 3.4. Sensing of Dopamine

**Figure 6:**
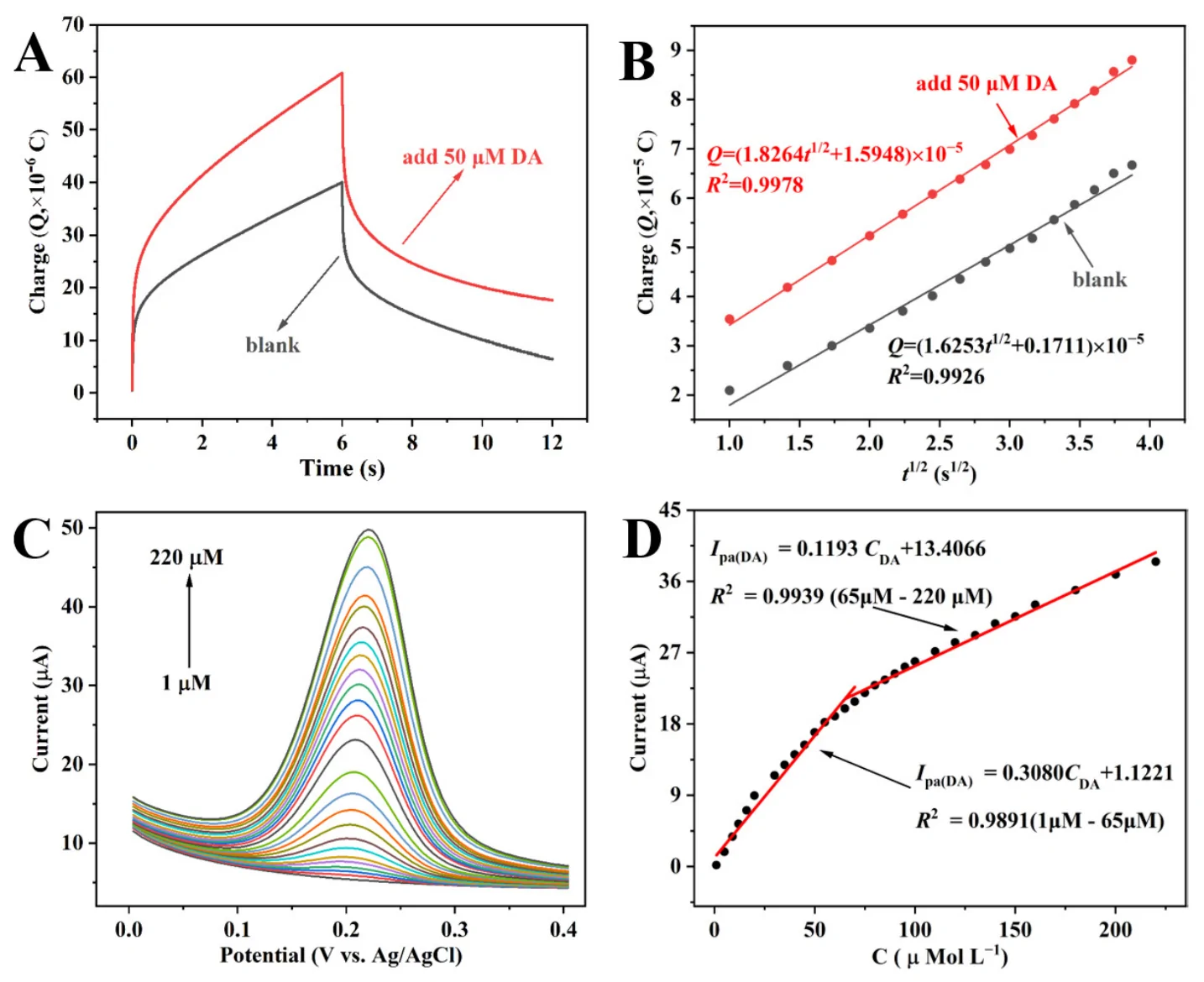
(**A**) CC curves of Fe@N-S-O-C/GCE before and after the addition of DA. (**B**) Linear fitting plot of charge number *Q* and the square root of response time *t*^1/2^ before and after the addition of DA. (**C**) DPV curves in the presence of different concentrations of DA. (**D**) Linear fitting plot of peak anodic current intensity (*I*_pa_) and DA concentration (*C*_DA_).

Chronocoulometric (CC) measurements were performed using the Fe@N-S-O-C/GCE in the absence and presence of 50 μM dopamine (DA) to further characterize its electrochemical behavior. The resulting chronocoulometric curves are shown in Figure 6A. Figure 6B shows a strong linear relationship was observed between the charge (*Q*) and the square root of time (*t*^1/2^), yielding the regression equation *Q* = (1.8264*t*^1/2^ + 1.5948) × 10^−5^ (*R*^2^ = 0.9978). This linear relationship is consistent with Anson’s equation [49]:

*Q* = 2*nFAD*^1/2^*C*π^1/2^*t*^1/2^ + *Q*_dl_ + *Q*_ads_ (5)

where *A* is the effective electroactive surface area, *D* is the diffusion coefficient, *C* is the concentration of DA, and *Q*_dl_ and *Q*_ads_ represent the double-layer charge and faradaic charge from adsorbed species, respectively. By substituting the slope from the linear regression into Anson’s equation (where *C* = 50 μM), the diffusion coefficient (*D*) of DA was calculated to be 7.67 × 10^−7^ cm^2^/s.

Quantitative analysis of DA was carried out via DPV (Figure 6C). The anodic peak current (*I*_pa_) exhibited a progressive increase upon successive concentration increments of DA from 1 to 220 μM, after which the signal reached a steady-state plateau. As presented in the calibration profiles (Figure 6D), two distinct linear regions were observed. Within the lower concentration range of 1–65 μM, the regression equation was *I*_pa_(μA) = 0.308*C*_DA_ − 1.122 (*R*^2^ = 0.9891), yielding a sensitivity of 4.357 μAμM^−1^ cm^−2^. In the higher concentration range of 65–220 μM, the equation was *I*_pa_(μA) = 0.119*C*_DA_ − 13.406 (*R*^2^ = 0.9939), with a corresponding sensitivity of 1.685 μAμM^−1^ cm^−2^. The sensitivities were calculated using the unrounded slopes of the calibration curves and normalized to the geometric area of the 3-mm-diameter GCE (0.0707 cm^2^), rather than to the electrochemically active surface area (ECSA). The limit of detection (LOD) was calculated to be 40 nM using the equation LOD = 3*σ/S*, where *σ* is the standard deviation of the current responses obtained from ten replicate measurements of blank PBS under identical experimental conditions, and *S* is the slope of the calibration curve in the lower-concentration range. These results demonstrate that the Fe@N-S-O-C/GCE sensor possesses a wide linear range and exceptional sensitivity for DA detection.

As summarized in Table 1, the Fe@N-S-O-C/GCE-based DA sensor exhibited two linear response ranges of 1–65 and 65–220 μM and a low LOD of 40 nM. Compared with the representative sensors reported in recent years, the proposed sensor offered a favorable balance between linear range and detection limit. Moreover, its satisfactory repeatability (RSD = 2.5%, *n* = 6) and reproducibility (RSD = 3.8%, *n* = 3) indicate reliable analytical performance.

**Figure 7:**
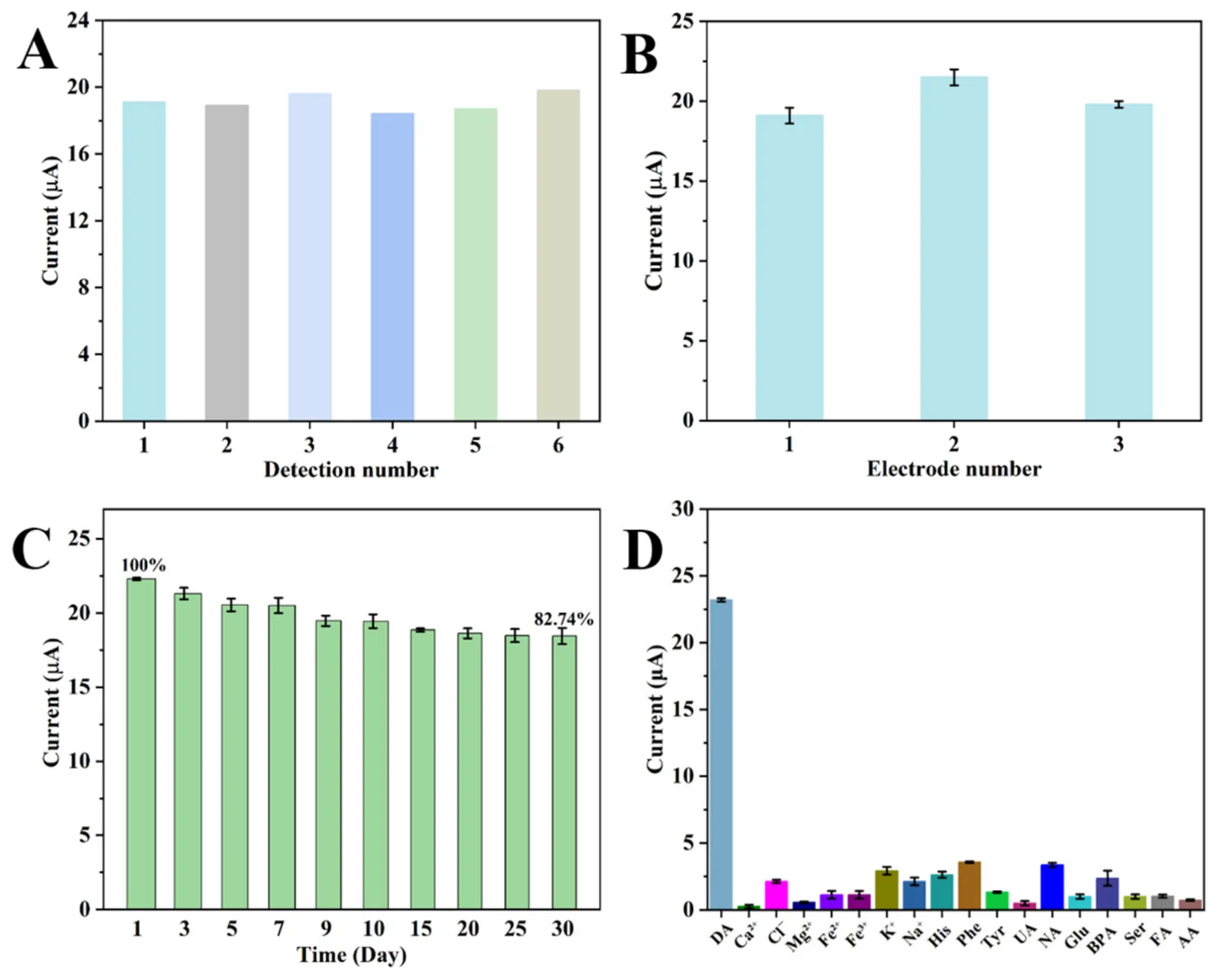
(**A**) Histogram of peak current intensities from six consecutive DPV detections. (**B**) Reproducibility test for the preparation of Fe@N-S-O-C/GCE (*n* = 3). (**C**) Influence of storage duration on the detection performance of Fe@N-S-O-C/GCE. (**D**) Current responses of Fe@N-S-O-C/GCE toward different interfering substances.

The response of the Fe@N-S-O-C/GCE toward 20 μM DA was evaluated by six consecutive DPV measurements. As shown in Figure 7A, the peak current remained nearly constant, with an RSD of 2.5%, indicating excellent short-term repeatability. To assess the reproducibility of the fabrication process, three independent Fe@N-S-O-C/GCE electrodes were prepared under identical conditions, and each electrode was tested three times. The results in Figure 7B showed an RSD of 3.8% for the peak current response to 20 μM DA, demonstrating the excellent reproducibility of the electrode preparation. The long-term stability of the Fe@N-S-O-C/GCE was evaluated using the same electrode throughout the 30-day storage period, with DPV responses recorded at fixed intervals under identical conditions. As shown in Figure 7C, the peak current remained at 82.74% of its initial value, demonstrating satisfactory long-term storage stability. The anti-interference performance of the Fe@N-S-O-C/GCE was also evaluated under optimized conditions. As shown in Figure 7D, various potentially interfering species, including metal ions (Na^+^, Cl^−^, K^+^, Ca^2+^, Mg^2+^, Fe^3+^, and Fe^2+^) and biomolecules (AA, UA, Glu, His, Phe, NA, BPA, Ser, FA, and Tyr), at concentrations 100 times higher than that of DA (i.e., 2 mM), caused negligible interference with the DA response. Meanwhile, the responses of these interferents were minimal, further demonstrating the excellent selectivity of the Fe@N-S-O-C/GCE for DA detection.

### 3.5. Real-Sample Analysis

The applicability for DA determination in pork was assessed by the standard addition method. Following pretreatment, the pork samples were spiked with DA at concentrations of 5, 20, and 200 μM and analyzed by differential pulse voltammetry (DPV). As summarized in Table 2, recoveries ranging from 95.97% to 106.73% were obtained, demonstrating the satisfactory accuracy of the proposed method and its potential for DA determination in pork samples.

Although these spike-recovery results support the applicability of the sensor to pork samples, further studies using well-characterized meat samples containing different levels of endogenous DA are required for a more comprehensive evaluation of its performance in complex food matrices. The proposed sensor may provide a promising platform for the rapid determination of DA in food-quality-control applications, and its integration with portable electrochemical workstations could further facilitate on-site monitoring. Nevertheless, before commercial or industrial implementation, additional efforts are needed to develop scalable and cost-effective material-synthesis methods, standardize electrode-fabrication procedures, improve long-term operational stability, and validate the sensor using a broader range of real food samples.

## 4. Conclusions

In summary, a novel enzyme-free electrochemical sensor based on an Fe@N-S-O-C nanocomposite was successfully developed for the rapid and sensitive detection of DA. The Fe@N-S-O-C nanomaterial was synthesized via a facile high-temperature calcination process using melamine and ferrous sulfate as precursors. Under the optimized sensing conditions, the Fe@N-S-O-C/GCE exhibited two linear response ranges of 1–65 and 65–220 μM, with a detection limit of 40 nM. In addition, the sensor demonstrated excellent analytical performance, including high reproducibility, good repeatability, satisfactory long-term storage stability, and strong anti-interference capability toward common coexisting species. Furthermore, its practical applicability was validated by recovery rates of 95.97–106.73% in pork samples. Nevertheless, a limitation of the present work is that the sensing performance was evaluated using a conventional glassy carbon electrode under laboratory conditions, while the integration of Fe@N-S-O-C into practical sensing devices has not yet been fully explored. Future work will therefore focus on developing integrated and portable electrochemical sensing platforms and evaluating their performance under practical conditions to facilitate on-site DA detection. Overall, this work provides an effective platform for DA detection and demonstrates the promising potential of iron-based nanocomposites in electrochemical sensing and food safety analysis.

## Figures and Tables

**Table 1:** Analytical characteristics of the Fe@N-S-O-C/GCE and representative electrochemical DA sensors.

Electrode	Detection Method	Linear Range (μM)	Sensitivity (μAμM^−1^cm^2^)	LOD (nM)	Repeatability, RSD (%)	Reproducibility, RSD (%)	Ref.
PANI	CV	0.15625–5	153.67	64	\	\	[50]
ZnO/GCE	SWV	0.6–12	28.8172	30	3.36 (*n* = 5)	3.42 (*n* = 5)	[51]
GCE/GO-AuNCs	DPV	1–25	30.3	28	\	\	[52]
D-PdMo/GCE	DPV	0.05–100	\	29	\	2.63 (*n* = 6)	[53]
V–CuO/GO/GCE	CV	10–100	\	8000	\	\	[54]
Au@SiO_2_-APTES/GCE	DPV	0.047–0.125 0.125–0.875	\	14	2.4 (*n* = 5)	3.1 (*n* = 6)	[55]
GOQDs-8/GCE	AMP	0.1–30	0.88	506	\	\	[56]
IL-MXene/GPE	DPV/Amperometry	10–2000	2.16	702	2.3 (*n* = 10)	1.9 (*n* = 8)	[57]
GCE-PPI-AuNPs	SWV	0.1–1.25	\	160	\	2.81 (*n* = 3)	[58]
Lc/CDs/GCE	DPV	0.25–9.82 9.82–76.81	\	80	\	\	[59]
Fe@N-S-O-C/GCE	DPV	1–65 65–220	4.357 1.685	40	2.5 (*n* = 6)	3.8 (*n* = 3)	This work

Note: PANI: Polyaniline; ZnO: Zinc Oxide; GO: Graphene Oxide; AuNCs: Gold Nanocrystals; D-PdMo: defect-engineered PdMo metallene; Au: Gold; SiO_2_: Silicon Dioxide; APTES: 3-Aminopropyltriethoxysilane; GOQDs: Graphene Oxide Quantum Dots; IL: Ionic Liquid; MXene: Two-Dimensional Transition Metal Carbide; GPE: Graphite Pencil Electrode; PPI: Poly(propylene imine); Lc: Laccase; CDs: Carbon Dots.

**Table 2:** Recovery of DA in pork samples determined using the Fe@N-S-O-C/GCE sensor.

Concentration Added (µM)	Concentration Calculation (µM)	Recovery (%)	RSD (%)
5	4.798 ± 0.325	95.97 ± 6.49	6.77
20	19.517 ± 0.375	97.58 ± 1.87	1.92
200	213.468 ± 1.465	106.73 ± 0.73	0.69

Note: Data are presented as mean ± SD (*n* = 3).

## Data Availability

The original contributions presented in the study are included in the article/Supplementary Materials. Further inquiries can be directed to the corresponding authors.

## Supplementary Materials

The following supporting information can be downloaded at: https://www.mdpi.com/article/10.3390/foods15162886/s1, Figure S1: displays the corresponding survey and high-resolution XPS spectra belonging to the Fe@N-S-O-C nanocomposite.
