# Supplementary material for: A High-Performance Fe@N-S-O-C Nanocomposite-Based Electrochemical Sensor for Dopamine Detection in Pork Samples
Source: Foods. 2026 Aug 18;15(16):2886. doi: 10.3390/foods15162886 (PMC13512036; doi:10.3390/foods15162886)
Supplement: Supplementary file 1 [file foods-15-02886-s001.zip › foods-4460244-supplementary.pdf]

Supplementary Materials

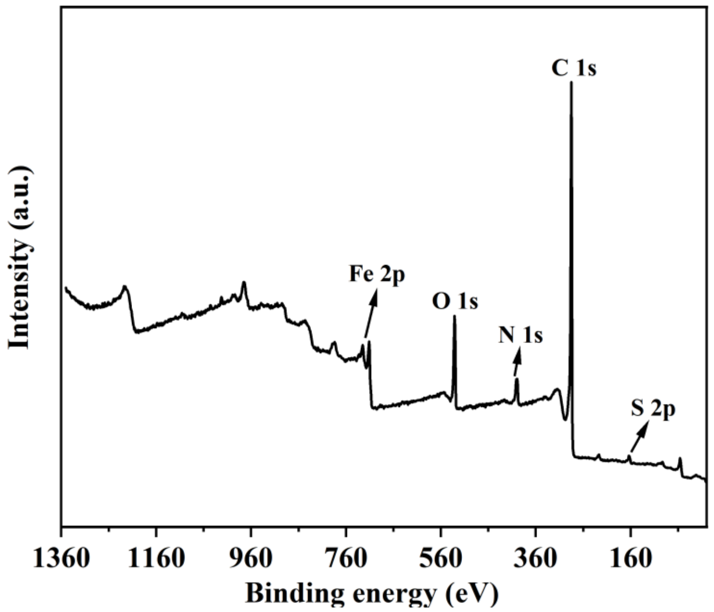

Figure S1 large-scale and high-resolution XPS spectra of Fe@N-S-O-C nanocomposites.
